# Immunoregulatory Effects of *Codonopsis pilosula* Polysaccharide Modified Selenium Nanoparticles on H22 Tumor-Bearing Mice

**DOI:** 10.3390/foods13244073

**Published:** 2024-12-17

**Authors:** Yan Long, Hongfei Ji, Jiajing Yang, Haiyu Ji, Keyao Dai, Wenjie Ding, Guoqiang Zheng, Juan Yu

**Affiliations:** 1 Yantai Key Laboratory of Characteristic Agricultural Bioresource Conservation & Germplasm Innovative Utilization, School of Life Sciences, Yantai University, Yantai 264005, China; 18282780162@163.com (Y.L.); hongfei10052024@163.com (H.J.); yangfan2812@163.com (J.Y.); haiyu11456@163.com (H.J.); 2 College of Food Science and Engineering, Tianjin University of Science and Technology, Tianjin 300457, China; dai13389086120@163.com (K.D.); dingwenjie1120@163.com (W.D.)

**Keywords:** *Codonopsis pilosula* polysaccharide (CPP), selenium nanoparticles (SeNPs), immunomodulatory activity, antitumor effects in vivo, H22 tumor-bearing mice, mitochondrial apoptosis pathway

## Abstract

*Codonopsis pilosula* polysaccharide (CPP) and rare element selenium (Se) have been proved to exert various biological activities, and our previous study demonstrated that selenium nanoparticles modified with CPP (CPP-SeNPs) possessed significantly enhanced tumor cytotoxicity in vitro. This study aimed to investigated the inhibitory effects of CPP-SeNPs complex on H22 solid tumors via immune enhancement. In this study, the H22 tumor-bearing mice model was constructed, and the potential mechanisms of CPP-SeNPs antitumor effects were further explored by evaluating cytokines expression levels, immune cells activities and tumor cells apoptotic indicators in each group. The results demonstrated that CPP-SeNPs effectively exerted dose-dependent protective effects on the immune organs of tumor-bearing mice in vivo, leading to increase in peripheral white blood cell counts and inhibition of solid tumor growth with inhibitory rate of 47.18% in high-dose group (1.5 mg/kg). Furthermore, CPP-SeNPs treatment significantly elevated the levels of TNF-α, IFN-γ, and IL-2 in mice sera, enhanced NK cell cytotoxicity, augmented macrophage phagocytosis capacity, as well as increased both the amounts and proliferation activity of lymphocyte subsets. CPP-SeNPs improved the immune system’s ability to clear tumor cells by up-regulating Bax expression while down-regulating Bcl-2 expression within solid tumors, indicating the potential activation of mitochondrial apoptosis pathway. Therefore, CPP-SeNPs administration can effectively inhibit tumor growth by enhancing immune response in tumor-bearing mice, which might be relevant to the regulation of gut microbiota short-chain fatty acids metabolisms. These findings could provide theoretical support and data foundation for further development of CPP-SeNPs as functional food and drug adjuvants.

## 1. Introduction

*Codonopsis pilosula* is a widely recognized plant with medicinal and edible properties, containing various bioactive compounds, including polysaccharides, polyphenols, flavonoids, sterols, alkaloids, and amino acids, and has demonstrated immunomodulatory, antioxidant, lipid-lowering activities, etc. [1,2]. Among these components, the polysaccharide compounds are particularly noteworthy [3]. Currently, researchers have primarily focused on extracting *Codonopsis pilosula* polysaccharides using hot water extraction and ethanol precipitation methods. However, there is limited information available regarding the presence of alcohol-soluble supernatants containing polysaccharides [4]. Selenium (Se) is an essential trace element that plays a crucial role in cancer prevention and inducing apoptosis in cancer cells [5,6]. Nanotechnology has led to the discovery of nano-selenium (SeNPs), which serve as a new source of selenium with potent biological activity and low toxicity [7,8]. Nevertheless, SeNPs tend to be unstable in nature and prone to aggregation [9]. By utilizing natural polysaccharides as stabilizers and modifiers in the preparation of SeNPs complexes, the advantages of polysaccharides and SeNPs can be combined to make the complexes better exert anti-tumor effects through cyclin regulation or targeting mitochondria [10,11], which holds great potential for expanding their applications in the food and pharmaceutical industries as dietary supplements.

Liver cancer is one of the leading cause of death from cancer worldwide, being serious threat to human physical and mental health [12]. Due to its initial lack of noticeable symptoms, liver cancer often goes undetected until it reaches advanced stages [13]. Unfortunately, by that time, surgical removal or transplantation options are limited, resulting in a general survival period of only about 6–20 months after diagnosis [14]. Numerous researchers have dedicated their efforts to combatting liver cancer over the past few decades [15]. Traditional chemotherapy drugs exert simultaneous toxic effects on both tumor cells and immune cells, potentially leading to the development of acquired drug resistance in tumor cells and compromising the therapeutic efficacy [16]. Tumor immunotherapy mainly including active immunotherapy and passive immunotherapy has become a research hotspot in the field of cancer treatment, which treats cancer by enhancing the ability of the body’s immune system to identify and attack cancer cells and has the characteristics of targeted attack on tumors and less toxic side effects [17]. *Codonopsis pilosula* polysaccharide (CPP), as the main active component, also possesses the potential to exert regulatory roles in the process of antitumor immunity [18]. Furthermore, advancements in nanotechnology have revolutionized early detection and treatment methods for various cancers. Functionalized nanoparticles offer advantages like smaller size and special modifications that enhance tumor targeting capabilities [19,20]. By harnessing the benefits of carbohydrates as natural active ingredients alongside nanotechnology applications, new possibilities emerge for preventing and treating liver cancer.

In our previous study, the *Codonopsis pilosula* polysaccharide modified selenium nanoparticles (CPP-SeNPs) had been successfully synthesized, exhibiting excellent stability and an average particle size of 75 nm, which effectively induced HepG2 cells apoptosis dose-dependently through classical mitochondrial signaling pathway [21]. However, there are currently no available reports on the inhibitory activity and action mechanisms of tumor cells proliferation in vivo. In this study, the H22 tumor-bearing mice model was established, and the effects of CPP-SeNPs complex on cytokines expression levels, immune cells activities and tumor cells apoptotic indicators were studied, which would further promote the further practical applications of CPP-SeNPs in food and drug industries.

## 2. Materials and Methods

### 2.1. Materials and Reagents

RPMI 1640 medium, Neutral Red, MTT, Cyclophosphamide, Con A, and LPS were purchased from Beijing Solarbio Science & Technology Co., Ltd. (Beijing, China). Antibodies to apoptosis regulator Bax, apoptosis regulator Bcl-2, and β-actin were provided by North American ImmunoWay Biotechnology Company (Plano, TX, USA). PE Anti-Mouse CD4 Monoclonal Antibody, FITC Anti-Mouse CD8 Monoclonal Antibody, FITC Anti-Mouse CD3 Monoclonal Antibody and PE Anti-Mouse CD19 Monoclonal Antibody were purchased from BioLegend Company (San Diego, CA, USA).

### 2.2. Synthesis of CPP-SeNPs

The *Codonopsis pilosula* powder was immersed in distilled water under following conditions: liquid-solid ratio of 40 mL/g, extraction time of 60 min, ultrasonic power of 370 W, and extraction temperature of 60 °C. The aqueous extracts were collected, concentrated, and precipitated with 4 volumes of ethanol. Then the supernatant was further concentrated using rotary evaporator, dialyzed with distilled water (MWCO of 600 Da), and purified using sephadex-G25 column to obtain CPP [21].

**Figure 1:**
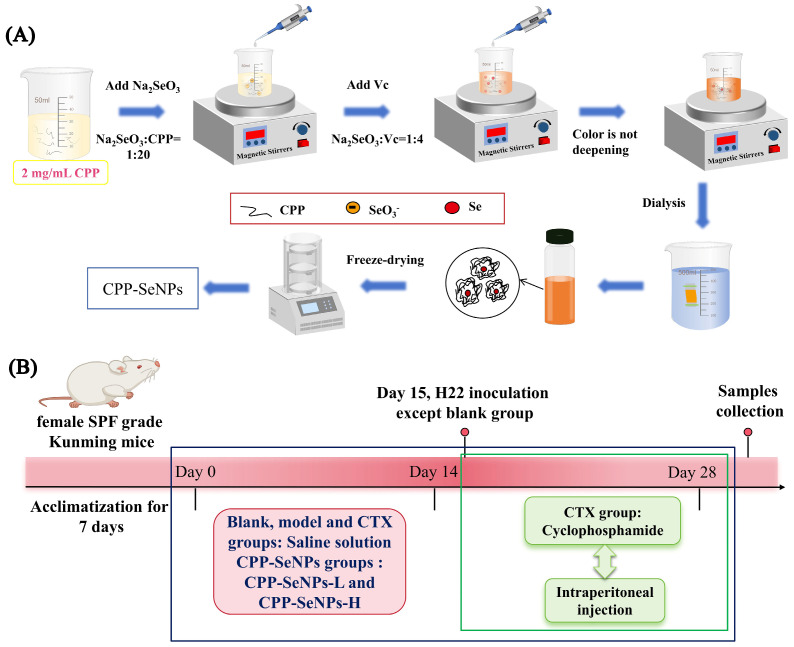
CPP-SeNP synthesis process (**A**) and animal experiment arrangement (**B**).

The redox reaction system of selenite and ascorbic acid was used to prepare CPP-SeNPs complexes [22], and the procedures were illustrated in Figure 1A. First, CPP and Na_2_SeO_3_ solutions were mixed under magnetic agitation (Na_2_SeO_3_ to CPP mass ratio of 1:20). Then, at room temperature and in the absence of light, the ascorbic acid solution was added and the reaction proceeded until the reaction solution turned red and reached the maximum intensity. After completion, the reaction solution was subjected to dialysis (molecular weight cut-off of 1000 Da) for 3 days. Finally, the CPP-SeNPs complex was obtained through freeze-drying.

### 2.3. Animal Experiment Design

Fifty SPF-grade Kunming mice (female, 8 weeks old) were procured from SPF (Beijing) Biotechnology Co., Ltd., under the license number SYXK (Beijing) 2019-0010. The mice were housed in the animal facility of Tianjin University of Science and Technology, operating under the license number SYXK (Jin) 2018-0001, maintaining an environment with relative humidity ranging between 45% and 55%, temperature maintained at 20–25 °C, and a light-dark cycle of 12 h each. Throughout the rearing period, ad libitum access to food and water was provided for all mice.

As presented in Figure 1B, after one week of acclimatization, the mice were randomly allocated into five groups (10 mice/group): (1) blank group (0.9% saline, 0.2 mL/d); (2) model group (0.9% saline, 0.2 mL/d); (3) CTX group (cyclophosphamide, 30 mg/kg/d) [23]; and two CPP-SeNPs treatment groups: low-dose group (0.75 mg/kg/d, SeNPs), and high-dose group (1.5 mg/kg/d, SeNPs). For mice in the blank, model and CTX groups, gavage administration with 0.2 mL of 0.9% saline was performed once a day for two weeks, while mice in the low and high dose groups received equal volume of CPP-SeNPs solution. On day 15, H22 hepatocellular carcinoma cells (1 ×10^7^ cells/mL, 0.2 mL) were inoculated into the axilla of right forelimb for all mice except those in the blank group, and the gavage administration was continued for another two weeks. At the same time, the CTX group was intraperitoneally injected with CTX solution until the end of experimental period [24].

### 2.4. Physiological Indices Collection of Mice

The mice weights of each group were measured and recorded before and after the experiment. Subsequently, the mice were killed by cervical dislocation, and the tumors, thymuses and spleens were quickly collected, weighed and recorded. Tumor inhibition rate (%) = (M1 − M2)/M1 × 100, where M1 represented the average tumor weight (g) of mice in model group and M2 represented the average tumor weight (g) of mice in experimental groups. Thymus or spleen indices were determined as thymus or spleen weights to the body weights ratios (mg/g).

### 2.5. White Blood Cells and Serum Cytokine Levels Determination in Mice

A volume of 100 μL fresh whole blood was collected from mice and rapidly added to microcentrifuge tubes containing anticoagulant (K_2_EDTA, 3 mg/mL, 7.5 μL). After thorough mixing, the leukocyte counts in each group were immediately determined using a XFA-6130 automatic blood analyzer (Nanjing, China). The expression levels of sera cytokines (IL-2, TNF-α, and IFN-γ) were determined following the instructions provided with the corresponding ELISA assay kits.

### 2.6. Splenic Lymphocytes Proliferative Activity Assay

The mitogenic effects of ConA and LPS on T and B lymphocytes in spleens were evaluated to determine their proliferative activities. In brief, the sterile suspension of splenic cells was prepared with the density of 1 × 10^7^ cells/mL, and inoculated into a 96-well plate (100 µL/well). The control group received 100 µL of RPMI 1640 medium per well, while the experimental groups received 100 µL solution (ConA, 5 μg/mL; or LPS, 10 μg/mL) per well. After incubation in 5% CO_2_ at 37 °C for 48 h, each well was treated with MTT solution followed by DMSO solution after additional 4 h. The absorbance values (OD) at 570 nm were measured using a BioRad 680 microplate reader (Hercules, CA, USA). The lymphocytes stimulation indices were calculated as the ratios of OD_1_ to OD_2_, (where OD_1_ represented the OD values for experimental groups and OD_2_ represented the OD values for control group).

### 2.7. Splenic NK Cells Activity Assay

The MTT assay was used to detect and evaluate splenic NK cell activties in each group. The splenic NK cells were isolated following the instructions provided with the mouse splenic NK cell isolation kit, and subsequently suspended in RPMI 1640 medium with the density adjusted to 2 × 10^6^ cells/mL, while H22 cells with an equivalent density were used as target cells. Experimental group: 100 μL of NK cells and 100 μL of H22 cells additions; effector cell control group: 100 μL of NK cells and 100 μL of RPMI 1640 medium additions; target cell control group: 100 μL of H22 cells and 100 μL of RPMI 1640 medium additions. Following incubation in a constant temperature incubator (5% CO_2_, 37 °C) for 48 h, MTT and DMSO solutions were introduced into the wells, followed by measurement of OD values at 570 nm. The formula used to calculate NK cell killing activity is as follows: NK cell killing activity = (1 − (OD_1_ − OD_2_)/OD_3_) × 100. Where OD_1_, OD_2_, OD_3_ were the OD values of experimental group, effector cell control group and target cell control group, respectively.

### 2.8. Peritoneal Macrophages Phagocytosis Assay

The neutral red solution were employed to assess and analyze the phagocytic activity of peritoneal macrophages in each group. Peritoneal macrophages from mice were collected under sterile conditions, resuspended in RPMI 1640 medium at a density of 5 × 10^6^ cells/mL, and then inoculated into a 96-well plates (100 µL/well). These plates were incubated in a constant temperature incubator (37 °C, 5% CO_2_) for 24 h, then the neutral red solution (*w*/*v*, 0.075%) was supplemented (100 μL/well). After 2 h incubation, the supernatant was removed and the wells were washed by PBS solution. Subsequently, cell lysate (anhydrous ethanol and acetic acid, *v*:*v*, 1:1) was added (100 μL/well), and then the absorbance were measured at 550 nm to evaluate the phagocytic ability of peritoneal macrophages.

### 2.9. Lymphocyte Subsets Distributions Assay

A total of 100 μL blood was collected from each mouse and added to two tubes containing 500 μL red blood cell lysate. In one tube, 2 μL FITC-CD19^+^ and PE-CD3^+^ antibodies were added, while in the other, 2 μL PE-CD4^+^ and FITC-CD8^+^ antibodies were added. These tubes were incubated for 20 min away from light, followed by removing the supernatant after centrifugation. The red blood cells were repeatedly lysed once, then washed with PBS twice, then re-suspended in 500 μL PBS solution, and the proportions of lymphocyte subsets in peripheral blood in each group was detected and analyzed by a flow cytometry (Bergen, NJ, USA).

### 2.10. H&E Staining Assay of Solid Tumors

H&E staining, or hematoxylin-eosin staining, is a common staining method used to distinguish between nuclei and cytoplasm [25]. The suspension of solid tumor cells was prepared and adjusted to a density of 1 × 10^7^ cells/mL. Subsequently, 10 μL suspension was placed on a slide and fixed with 4% paraformaldehyde. Then the solid tumor cells from different treatment groups were stained by hematoxylin and eosin, observed and photographed under an inverted fluorescence microscope (Nikon, Tokyo, Japan).

### 2.11. Western Blot Detection of Solid Tumors

The expressions of apoptotic proteins in different groups were detected using Western blot method. Solid tumors from mice in each group were collected and cell lysate containing 1% PMSF was added. After being centrifuged at 12,000 rpm for 10 min under 4 °C, the supernatant was collected for SDS-PAGE gel electrophoresis. The proteins were subsequently transferred onto a polyvinylidene fluoride (PVDF) membrane using the semi-dry transfer technique and sealed with 5% BSA. Following that, Bax and Bcl-2 antibodies were subjected to an incubation period of 1.5 h at room temperature, respectively. Subsequently, the secondary antibody labeled with horseradish peroxidase corresponding to the primary antibody was introduced and allowed to incubate for another 1.5 h at room temperature. Finally, the enhanced ECL chemiluminescent substrate kit was used to display the target proteins on the film, and protein bands on the films were captured using Quantity One 4.6.2 software, and the grayscale values of each band were determined by Image J v1.51 software (National Institutes of Health, Bethesda, MD, USA).

## 3. Results and Discussion

### 3.1. Physiological Index Collection Results of Tumor-Bearing Mice

**Figure 2:**
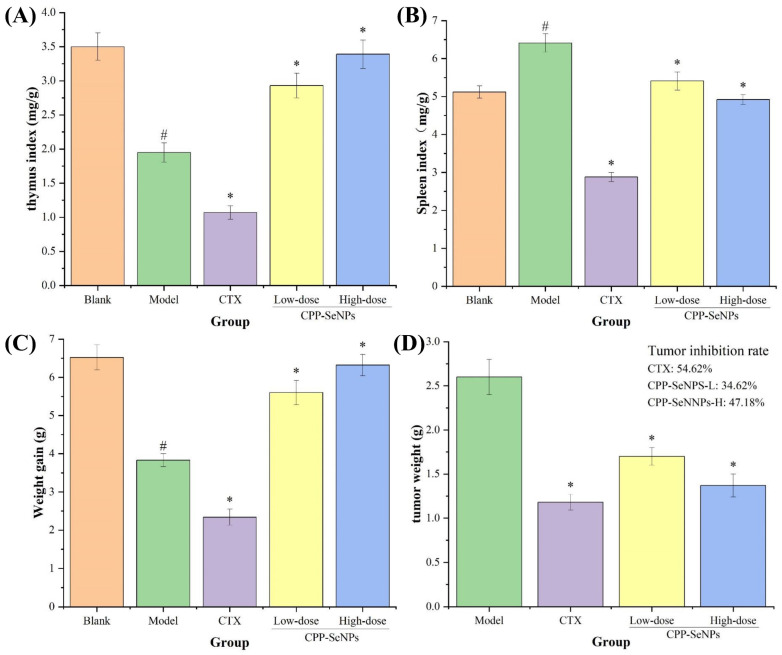
Effects of CPP-SeNPs on thymus indices (**A**), spleen indices (**B**), weight gain (**C**), and tumor weights (**D**) of H22-bearing mice. Note: ^#^, *p* < 0.05 compared with blank group; *, *p* < 0.05 compared with model group.

Body weight change is an important indicator of the growth rate of an organism, and body weight gain can be used to assess the toxicity of a certain biologically active compound to the organism [26]. Furthermore, the immune function of the body is closely associated with the thymus and spleen, and the ratio of these organs to body weight can serve as an indicator of the body’s immune strength to some extent [27]. Many studies have confirmed that tumor development is often accompanied by thymic atrophy and splenomegaly [28]. Therefore, in order to confirm the in vivo antitumor effects of CPP-SeNPs complexes and whether there are toxic side effects on the organism, H22 tumor-bearing mice model was established, and the changes in gained body weights, immune organs indices, tumor weights, and tumor inhibition rates of different treatment groups were investigated by gavage administration of CPP-SeNPs complexes, and the results were displayed in Figure 2.

Compared with blank group, the body weight growth rates and thymus indices of mice in model group were significantly decreased (*p* < 0.05), while the spleen indices were remarkably increased (*p* < 0.05), indicating that the solid tumors malignant growth seriously damaged the immune organ function of mice, resulting in thymus atrophy and spleen enlargement. Compared with model group, the weight gain and immune organ index of mice were obviously improved after the administration of CPP-SeNPs (*p* < 0.05), and the average tumor weights were significantly lower than that of model group (*p* < 0.05) dose-dependently. In addition, it could also be seen that the weight gain and immune organs indices of the high-dose CPP-SeNPs group were close to the levels of blank group, and the tumor inhibition rate reached 47.18%. In contrast, although CTX group presented a higher tumor inhibition rate (54.62%), it was found in the experiment that after CTX treatment, the mice showed loss of appetite, dry and dull fur, poor mental state, and immune organs indices were even lower than those in model group (*p* < 0.05), which confirmed that CTX showed obvious toxic and side effects on the body, leading to immune suppression, which was consistent with the previous results [29].

### 3.2. White Blood Cell Counts and Serum Cytokine Levels Results

**Figure 3:**
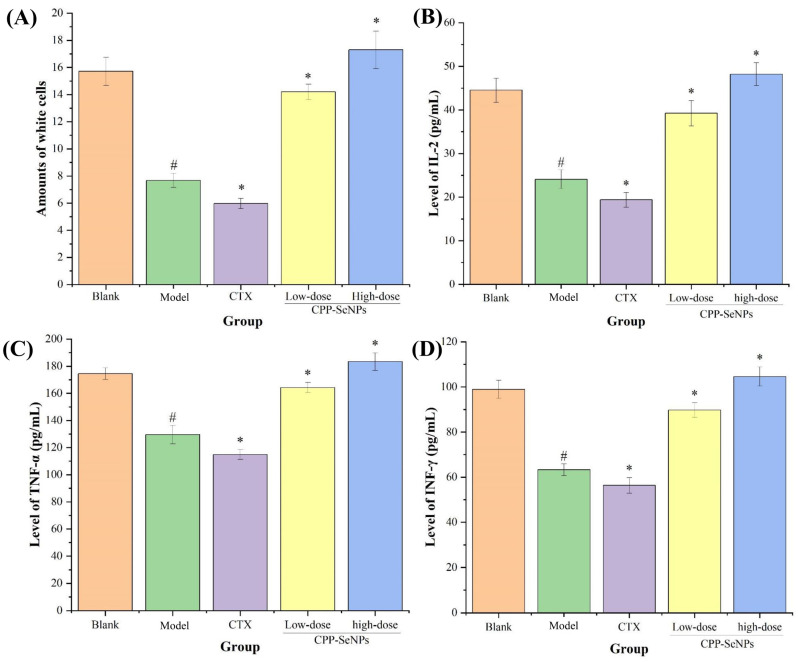
Effects of CPP-SeNPs on peripheral blood leukocytes (**A**), and IL-2 (**B**), TNF-α (**C**), and INF-γ (**D**) levels of H22-bearing mice. Note: ^#^, *p* < 0.05 compared with blank group; *, *p* < 0.05 compared with model group.

Cytokines, which are synthesized and secreted by both immune and non-immune cells upon stimulation, have significant functions in regulating the immune system and facilitating cellular proliferation [30]. TNF-α is an important regulator of inflammation and immune response, and is extremely important for the direct killing of tumor cells [31]. IL-2 plays a crucial role in augmenting the cytotoxicity of NK cells, facilitating the expansion and specialization of T and B cells, as well as stimulating antibody production. Additionally, it is classified alongside TNF-α within Th1 cells and actively contributes to cellular immune responses [32]. IFN-γ is a pleiotropic cytokine, mainly secreted by activated T lymphocytes, and has a strong immunomodulatory effect [33]. Therefore, changes in sera concentrations of TNF-α, IFN-γ, IL-2 can be used as important markers of cellular immune function. The number of peripheral blood leukocytes and the expression of three cytokines in mice sera of different groups were evaluated and analyzed, and the results were displayed in Figure 3. Compared with the blank group, the number of leukocytes and the levels of three cytokines were significantly reduced in model group (*p* < 0.05). CTX treatment significantly decreased the number of leukocytes and the levels of the three cytokines in the blood of mice (*p* < 0.05) compared with the model group; whereas, the number of leukocytes and the expression levels of sera cytokines were significantly increased after gavage of CPP-SeNPs (*p* < 0.05) in a dose-dependent manner. In addition, it could also be seen by comparative analysis of the experimental data that the above indices in the CPP-SeNPs high-dose-treated group were slightly higher than the levels of blank group, which indicated that CPP-SeNPs could effectively maintain leukocytes at a normal level and promote the secretion of cytokines, which presented good immunomodulatory activities.

### 3.3. Proliferative Activity Analysis of Splenic Lymphocytes

**Figure 4:**
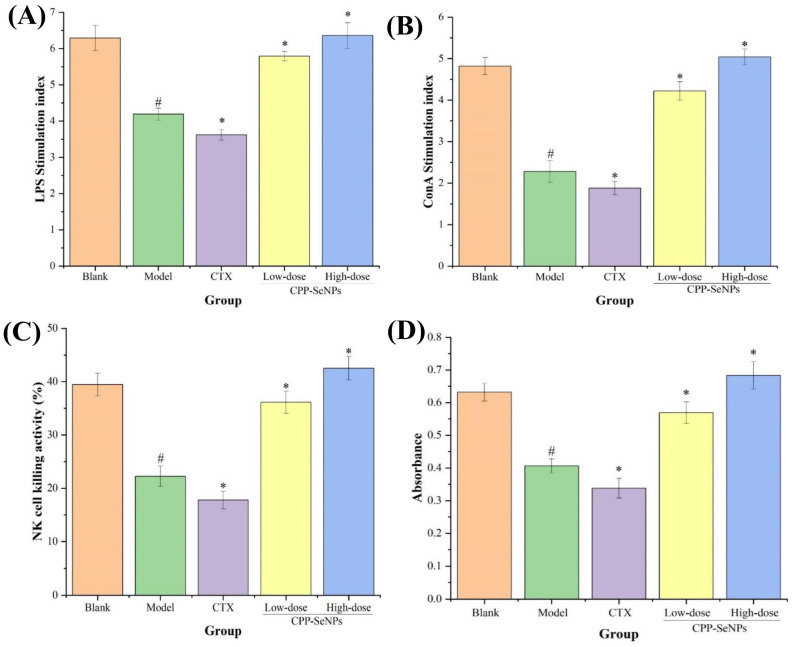
Effects of CPP-SeNPs on proliferative capacity of splenic B (**A**) and T (**B**) lymphocytes, killing capacity of splenic NK cells (**C**) and peritoneal macrophages phagocytosis (**D**) in mice. Note: ^#^, *p* < 0.05 compared with blank group; *, *p* < 0.05 compared with model group.

Lymphocytes play crucial roles in body’s immune system, and their proliferation capacities serves as direct indicators of immune function [34]. The spleen is an essential organ for immunity, housing numerous T and B lymphocytes. Typically, tumor growth and advancement coincide with the suppression of the organism’s immune response [35]. To evaluate the potential anti-hepatocellular carcinoma effects of the drug on immune enhancement, this study employed ConA and LPS for in vitro stimulation. The proliferative capacity of splenic lymphocytes from different treatment groups in tumor-bearing mice was compared and analyzed. As presented in Figure 4A,B, the stimulation indices of T and B cells in spleens of model group were obviously reduced compared with blank group (*p* < 0.05), which suggested that tumor growth had detrimental impacts on mice spleens, potentially impairing splenic lymphocytes functions and overall immune response. In contrast to the model group, both ConA-stimulated T-lymphocyte proliferation and LPS-stimulated B-lymphocyte proliferation were significantly decreased in mice treated with CTX (*p* < 0.05), indicating that CTX inhibited the body immunocompetence. Conversely, dose-dependent improvements were observed in splenic lymphocyte stimulation indices following oral administration of CPP-SeNPs (*p* < 0.05), confirming that combining CPP with SeNPs effectively stimulated the proliferative abilities of splenic lymphocytes in tumor-bearing mice, which could specifically enhance cellular immunity and humoral immunity responses against immunosuppression induced by tumor microenvironment.

### 3.4. Splenic NK Cells Killing Activity Analysis

NK cells are a subset of lymphocytes that play a crucial role in immune response by directly inducing cytotoxicity and secreting cytokines such as IFN-γ [36]. The killing activities of splenic NK cells in different treatment groups were presented in Figure 4C. The results demonstrated that the splenic NK cell killing activity was significantly decreased in model group (*p* < 0.05) compared with blank group, potentially due to impaired function caused by malignant proliferation of H22 hepatocellular carcinoma cells. Moreover, compared with model group, mice treated with CTX exhibited even lower splenic NK cells killing activity, indicating the detrimental effects on immune system. Conversely, CPP-SeNPs administration resulted in a dose-dependent enhancement trend in splenic NK cell killing activity, which was obviously ascended compared with model group (*p* < 0.05), suggesting that CPP-SeNPs could effectively enhance immunity against H22-bearing mice for eliminating H22 tumor cells via activating NK cells capacity.

### 3.5. Peritoneal Macrophage Phagocytic Activity Analysis

The macrophage, an essential component of the immune system, assumes critical functions in safeguarding the host from intruders like cancer cells and disease-causing microorganisms [37]. Macrophage phagocytosis is an essential element in maintaining body homeostasis, serving as a fundamental component of nonspecific immunity and laying the foundation for generating specific immune responses [38]. The phagocytic activity results of macrophages were shown in Figure 4D. Compared with blank group, mice in model group exhibited a significant reduction in abdominal macrophage phagocytic activity due to solid tumor infiltration (*p* < 0.05). The CTX group demonstrated significantly lower abdominal macrophage phagocytic activity compared with model group (*p* < 0.05) due to immune damage caused by CTX. In contrast, the mice treated with CPP-SeNPs showed a dose-dependent enhancement of macrophage phagocytic activity compared with model group, which was statistically significant (*p* < 0.05), which suggested that CPP-SeNPs could effectively enhance the macrophages mediated tumor cells clearance.

### 3.6. Analysis of Lymphocyte Expression in Peripheral Blood

**Figure 5:**
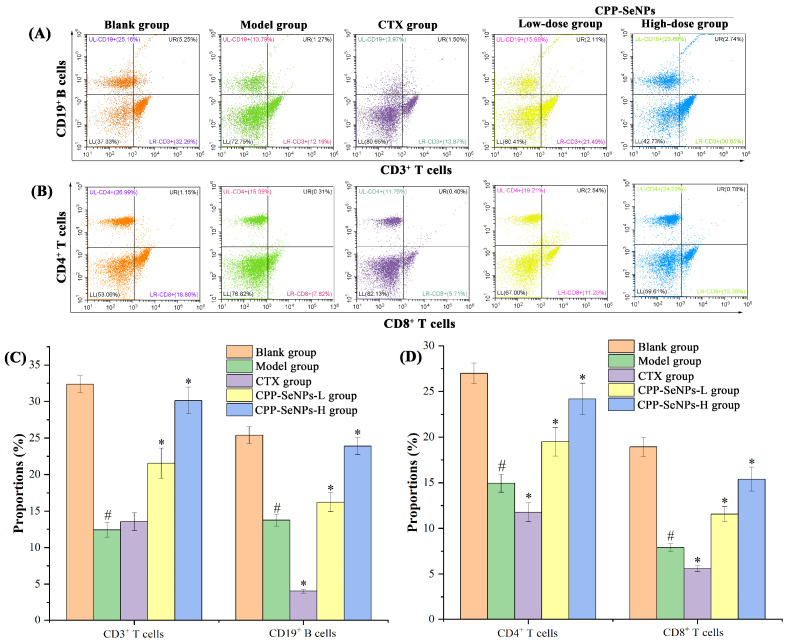
Effects of CPP-SeNPs on the distributions and proportions of lymphocytes subsets in peripheral blood. (**A**) CD3^+^ T cells and CD19^+^ B cells distributions; (**B**) CD4^+^ T cells and CD8^+^ T cells distributions; (**C**) CD3^+^ T cells and CD19^+^ B cells proportions; (**D**) CD4^+^ T cells and CD8^+^ T cells proportions. Note: ^#^, *p* < 0.05 compared with blank group; *, *p* < 0.05 compared with model group.

The circulatory system receives peripheral blood from hematopoietic organs, which contains mature immune cells that directly contribute to the body’s immunity and perform biological functions [39,40]. The CD3 molecule serves as a surface marker on T cells, facilitating the transmission of antigen recognition signals to these cells. Meanwhile, CD19 acts as a surface marker on B cells and plays a crucial role in promoting their recognition and response towards antigens. CD4 and CD8 are expressed on the surface of helper T lymphocytes and cytotoxic T lymphocytes, respectively, and play crucial roles in anti-tumor immune response [41,42,43]. Therefore, the proportions of lymphocyte subsets in the bloodstream were assessed and relevant results were presented in Figure 5.

As depicted, the proportions of CD3^+^ T cells, CD19^+^ B cells, CD4^+^ T cells and CD8^+^ T cells in the peripheral blood of model group were remarkably decreased (*p* < 0.05) compared with blank group, suggesting that solid tumors hindered both the quantity and activity of T cells and B cells in mice. Following intraperitoneal injection of CTX, there was a further reduction observed in lymphocyte subsets within the peripheral blood of tumor-bearing mice compared with those in model group (*p* < 0.05), which indicated that CTX presented detrimental impacts on the immune system. However, upon oral administration of CPP-SeNPs, it was found that tumor-bearing mice had dose-dependently higher proportions of T lymphocytes and B lymphocytes than those observed in model group (*p* < 0.05), which suggested that CPP-SeNPs could enhance both specific recognition ability towards tumors of CD4^+^ T cells and direct killing activity of CD8^+^ T cells, while effectively inhibiting transplanted solid tumor growth [44].

### 3.7. H&E Staining Results of H22 Solid Tumors

**Figure 6:**
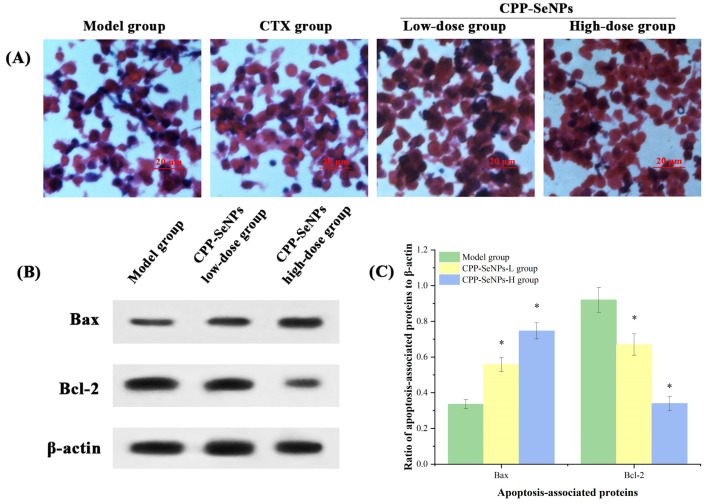
Effects of CPP-SeNPs administration on solid tumor cells condition. (**A**) H&E staining results; (**B**) western blot determination; (**C**) Bax or Bcl-2 expression ratios to β-actin. Note: *, *p* < 0.05 compared with model group.

The cells status in solid tumors of mice in each group was further analyzed using H&E staining [45], and the results were presented in Figure 6A. In model group, the solid tumor cells exhibited relatively intact cell morphology with uniform purple nucleus staining, indicating the good cells growth state. However, some abnormal cell morphologies may be attributed to nutritional deficiency caused by malignant proliferation of H22 cells in vivo or apoptosis induced by mechanical damage during single-cell suspension preparation. Compared with model group, higher proportions of apoptosis was observed in solid tumor cells of mice treated with CPP-SeNPs, which was mainly characterized by increased proportions of irregular and abnormal cells dyed red, possibly accompanied by nuclear lysis, which showed a dose-dependent relationship. Additionally, significant apoptosis was also observed in CTX group, indicating the direct cytotoxicity towards tumor cells. Therefore, it could be preliminarily inferred that intragastric administration of CPP-SeNPs could indirectly promote H22 cells apoptosis in vivo and thus achieve inhibition of solid tumor growth.

### 3.8. Western Blot Detection of Apoptosis in Solid Tumors

Bcl-2 family proteins are significant contributors to the process of programmed cell death, and the relationship between apoptosis and mitochondria can be observed by determining the expression levels of two apoptosis-related proteins, Bax and Bcl-2 [46]. To initially analyze the mechanism of action associated with apoptosis in mouse solid tumor cells, the expression of Bax and Bcl-2 proteins was determined by western blot method, and the results are shown in Figure 6B,C. As shown, after gavage of CPP-SeNPs, the expression levels of pro-apoptotic protein Bax in solid tumor cells of mice were significantly ascended in a dose-dependent manner (*p* < 0.05), while the expression levels of anti-apoptotic protein Bcl-2 were remarkably reduced (*p* < 0.05), suggesting that CPP-SeNPs induced apoptosis in H22 solid tumor cells might be related to mitochondrial dysfunction [47,48].

### 3.9. Possible Immunomodulatory Mechanism Analysis and Application Prospects

Exogenous chemicals, including polysaccharides with high molecular weights, are unable to penetrate the vascular barrier and have minimal contact with the body’s immune cells, which cannot directly exert immunomodulatory effects. The intricate relationship between the immune system and alterations in gut microbiota is undeniable [49]. As reported, polysaccharides often play critical roles in regulating the metabolism of intestinal microorganisms, and their final or intermediate metabolites can enhance the body’s immunity [50]. Previous research proved that *Codonopsis pilosula* polysaccharides could primarily improve *Limosilactobacillus* and *Muribaculum* abundance in digestive tracts of immunodeficient mice, and promote the short-chain fatty acids (mainly butyric acid) metabolism [51]. Besides, selenium-enriched yam glycoprotein exhibited similar immunomodulatory functions via NF-κb signaling pathway, which might be relevant to the enhancement of acetic acid metabolism [52].

In the present study, CPP-SeNPs were prepared using selenium nanoparticles modified via CPP, and presented strong immunoregulatory effects on various immune cells in tumor-bearing mice, which might be accomplished by regulating the short-chain fatty acids metabolism of intestinal flora [53]. As reported, the 3 primary short-chain fatty acids, namely acetate, propionate, and butyrate, are transported into cells via specific transporters, and have the potential to regulate various immune cells metabolism, thereby potentially reprogramming the tumor microenvironment [54]. Therefore, the investigation of interaction mechanisms between CPP-SeNPs-enhanced short-chain fatty acids metabolisms and immune cell activation will be the primary focus of our forthcoming researches.

With the increasing awareness of health among individuals, there is a growing urgency to enhance their own immune system. CPP modified selenium nanoparticles, as a kind of immunomodulator, have demonstrated significant potential in regulating immunity and inducing tumor cell apoptosis in mice with tumors through oral administration, thus offering extensive application prospects. In comparison to injectable thymopentin or vaccines, other immunostimulants, the oral form presents a wider range of applications with reduced risks [55,56]. CPP-SeNPs can be utilized for the development of immune-enhancing functional foods, such as yogurt, beverages, and dietary supplements food industry, while employed as therapeutic adjuvants for patients with tumors or immunodeficiency in pharmaceutical industry.

## 4. Conclusions

Polysaccharide-modified nano-selenium complexes lack the ability to traverse the vascular barrier, and the in vivo anti-tumor effects are not associated with the direct tumor cells inhibition. Therefore, the impacts of CPP-SeNPs on the immune system of H22 tumor-bearing mice were evaluated in this study. Results revealed that CPP-SeNPs oral administration effectively protected the immune organs of tumor-bearing mice and suppressed solid tumor growth with inhibitory rate of 47.18%. Furthermore, CPP-SeNPs significantly promoted NK cell cytotoxicity, macrophage phagocytic activity, as well as lymphocyte subsets proliferation and proportions in peripheral blood of tumor-bearing mice, finally leading to the apoptosis induction in solid tumor cells possibly due to mitochondrial membrane potential loss. Compared with the specific antibodies injection, protein vaccines immunization and immune cells modification in conventional tumor immunotherapy, the CPP-SeNPs exhibit a relatively weaker capacity for immune system activation through oral administration, while they could offer enhanced safety. Therefore, they are more suitable to play immune auxiliary roles as dietary adjuvants in the treatments of patients. However, further researches are still required to elucidate the immunomodulatory mechanisms of CPP-SeNPs through gut microbiota short-chain fatty acids metabolisms.

## Data Availability

The original contributions presented in this study are included in the article. Further inquiries can be directed to the corresponding authors.
